# Palbociclib in Combination with Endocrine Therapy in Patients with Metastatic Breast Cancer in a Real-World Population: Impact of Dose-Intensity, Dose Reductions and Cycle Delays on Efficacy

**DOI:** 10.3390/curroncol33010051

**Published:** 2026-01-15

**Authors:** Julie Coussirou, Julien Grenier, Alice Mege, Antoine Arnaud, Françoise De Crozals, Emmanuel Bonnet, Léa Vazquez

**Affiliations:** 1 Sainte Catherine, Institut du Cancer Avignon-Provence, 84918 Avignon, France; j.coussirou@isc84.org (J.C.); j.grenier@isc84.org (J.G.); a.mege@isc84.org (A.M.); a.arnaud@isc84.org (A.A.); f.decrozals@isc84.org (F.D.C.); 2 Clinique Beausoleil, 34080 Montpellier, France; emmanuel.bonnet.pro@gmail.com

**Keywords:** palbociclib, real-world, dose-intensity, metastatic breast cancer, progression-free survival

## Abstract

**Purpose**: With the addition of palbociclib to endocrine therapy, many hormone receptor-positive (HR+) metastatic breast cancer (mBC) patients experience toxicities that can lead to dose reductions and cycle delays. We examined the actual doses of palbociclib received by patients and their treatment responses. These dose adjustments, made at the physician’s discretion, are not always consistent with pharmaceutical company recommendations. The aim of this study was to assess the influence of dose adjustments on dose intensity and treatment response in our patients. **Methods**: Records of patients with HR+ mBC treated with palbociclib between December 2016 and January 2019 at the Sainte-Catherine Institute were retrospectively reviewed. Dose intensity was defined as the total dose of palbociclib received by each patient during the first six months of treatment. Anticipated dose reductions and extended cycle delays were recorded. Treatment response at six months and survival were assessed using statistical analyses. **Results**: A total of 131 women were included; the median age was 67 years. Forty-six patients (35%) experienced an anticipated dose reduction or an extended cycle delay during the first six months of treatment. Logistic regression analysis showed that factors correlated with six-month treatment response included anticipated dose reduction or extended cycle delay (OR = 14.6, 95% CI 3.74–97.4, *p* < 0.001), cycle delay > 4 weeks (OR = 5.94, 95% CI 1.58–21, *p* = 0.01), initial dosage < 125 mg (OR = 4.09, 95% CI 1.13–13.7, *p* = 0.034), and six-month dose intensity < 14,250 mg (OR = 26.0, 95% CI 4.91–481, *p* < 0.001). **Conclusions**: In this real-world assessment of clinical outcomes in French patients with HR+ mBC treated with palbociclib, a palbociclib dose intensity lower than recommended—particularly due to cycle delays longer than four weeks—was associated with an increased risk of six-month disease progression.

## Simple Summary

With the addition of palbociclib to endocrine therapy, hormone receptor-positive metastatic breast cancer patients may experience toxicities leading to dose reductions and cycle delays. We examined the actual dose received during the first six months of treatment and the patient response. Records of women treated at Sainte-Catherine Institute were retrospectively reviewed anticipated dose reductions, extended cycle delays, six-month dose intensity, and treatment response were analyzed. Among 131 patients, 35% experienced an anticipated dose reduction or extended cycle delay. Logistic regression showed that anticipated dose reduction or extended cycle delay, cycle delay longer than four weeks, an initial dosage below 125 mg, and a six-month dose intensity under 14,250 mg were strongly associated with six-month progression. In this real-world study, lower palbociclib dose intensity, particularly due to prolonged cycle delays, was associated with an increased risk of early disease progression.

## 1. Introduction

As the most prevalent cancer and the leading cause of cancer-related death among women worldwide, breast cancer constitutes a major public health issue [1]. In France, nearly 61,000 new cases of breast cancer were diagnosed and more than 12,000 deaths were recorded in 2023 [2]. Approximately 75% of breast tumors express the estrogen and/or progesterone receptor (hormone receptor-positive, HR+). In the metastatic setting, the standard-of-care first-line treatment for these patients is endocrine therapy alone or endocrine therapy combined with targeted therapy [3].

Endocrine therapy is the preferred option for hormone receptor-positive disease, even in the presence of visceral metastases (in non-visceral crisis setting), unless there is concern or evidence of endocrine resistance or a need for a rapid response [4]. Unfortunately, most patients treated with endocrine therapy eventually develop resistance, leading to disease progression [5]. Molecular targeted therapies such as palbociclib, a first-in-class potent oral inhibitor of cyclin-dependent kinases 4 and 6, have demonstrated improved progression-free survival compared with endocrine therapy alone in postmenopausal patients with HR+, HER2-recurrent or stage IV breast cancer [3,4].

Palbociclib, in combination with endocrine therapy, is generally considered safe and well tolerated [6]. However, for the management of adverse events in patients receiving palbociclib (CTCAE), a hematologic toxicity management algorithm has been recommended by the pharmaceutical company. This algorithm, mandatory in clinical trials, may not always be strictly followed in real-world clinical practice. In fact, some oncologists—or occasionally patients themselves—may choose to reduce doses or delay cycles outside the guidelines to improve treatment tolerance and quality of life. The most common adverse events of palbociclib are neutropenia and leukopenia, leading to intercurrent infections, anemia, fatigue, and nausea, all of which negatively affect quality of life [7]. When palbociclib is combined with endocrine therapy, grade 3–4 neutropenia occurs in approximately 65% of patients and can be managed by dose reduction, temporary interruption, or cycle delay. Permanent discontinuation due to neutropenia occurs in about 6% of patients [7].

To date, data evaluating the effectiveness of palbociclib combined with endocrine therapy come primarily from the PALOMA-2 and PALOMA-3 clinical trials [6,8]. In these studies, dose reductions and cycle delays performed according to the palbociclib management algorithm did not show any negative association with treatment response or survival [9]. However, it remains important to assess the impact of anticipated dose reductions or extended cycle delays on treatment efficacy. This would allow clinicians to make informed decisions to improve patients’ quality of life while remaining aware of the potential risk to efficacy.

Because of the limited real-world evidence, we conducted this retrospective analysis of patients receiving palbociclib under real-life conditions to better define the relationship between treatment dose intensity, dose reductions, and cycle delays and their impact on efficacy.

## 2. Materials and Methods

### 2.1. Patients and Study Design

The study was approved by the Avignon-Provence Cancer Institute Ethics Committee (approval number 2024-06) on 19 December 2024. We identified all patients treated with a combination of endocrine therapy and palbociclib for metastatic breast cancer (all treatment lines included) between 1 December 2016, and 1 January 2019, at our institution, using our clinical pharmacy database. Data were extracted from both electronic and paper medical records. Inclusion criteria were histologically confirmed metastatic breast cancer and at least three completed cycles of palbociclib therapy, or fewer than three cycles completed due to toxicity. A minimum follow-up period of six months after the first day of treatment was required.

The following data were collected: Eastern Cooperative Oncology Group (ECOG) performance status, age, weight, pretreatment blood cell count, cancer type, number of visceral and bone metastatic sites, and previous treatments. The start and end dates of palbociclib treatment, the metastatic treatment line, the starting dose, and the associated endocrine therapy were also recorded. Six-month dose intensity, the number of cycles completed within six months, cycle delays, and dose reductions were documented.

### 2.2. Endpoints

The main endpoint was to assess the impact of anticipated dose reductions or extended cycle delays on treatment dose intensity and six-month treatment response. Six-month treatment response was defined as the absence of disease progression (PD vs. no PD) at six months. For each patient, six-month dose intensity was calculated. Dose intensity was defined as the total dose of palbociclib (in milligrams) received during the first six months of treatment, starting from day 1 of cycle 1. Recommendations for the management of patients (cycle delays, dose reductions, or permanent discontinuation) while on palbociclib were based on the Pfizer prescribing information and the National Comprehensive Cancer Network (NCCN) guidelines (Table 1). Patients whose initial dose was reduced, who experienced an early dose reduction, an extended cycle delay, or a premature discontinuation outside toxicity management guidelines were considered to have received a non-optimized palbociclib dose intensity. Hematologic and non-hematologic toxicities were graded according to the National Cancer Institute Common Terminology Criteria for Adverse Events (CTCAE, version 4.3) [10]. As recommended, hematologic laboratory tests were performed on day 1 and day 15 for the first two cycles, and only on day 1 for all subsequent cycles [7].

### 2.3. Statistical Analysis

Statistical analyses were performed using R-4.1.1. All results were double-checked using R software (version 4.0.4).

Descriptive statistics were used to summarize baseline characteristics, presented as mean (standard deviation) and median (range) for continuous variables, and as counts (percentages) for categorical variables. Associations between categorical variables were assessed using the Pearson χ^2^ test or Fisher’s exact test, as appropriate, and associations between continuous variables were assessed using the two-sided *t*-test or the Wilcoxon rank-sum test. Statistical significance was defined as a *p*-value below 0.05.

Kaplan–Meier survival curves and log-rank tests were generated to assess and compare progression-free survival (PFS). PFS was defined as the time from treatment initiation to disease progression or death from any cause. Disease control, defined as complete response, partial response, or stable disease (CR/PR/SD), was used for descriptive purposes only. Median follow-up time was calculated using the inverse Kaplan–Meier method.

Multivariate logistic regression analyses were performed to identify independent predictors of six-month disease progression (PD vs. no PD). Considering the limited number of six-month progression events, the number of variables included in multivariate models was restricted to avoid overfitting. Multivariate analyses were therefore considered exploratory and hypothesis-generating. Receiver operating characteristic (ROC) curve analyses were performed to evaluate the ability of biomarkers to discriminate between patient populations.

## 3. Results

### 3.1. Patients

Between 2016 and 2019, a total of 131 female patients were included in this retrospective study. Most patients were managed within a multidisciplinary care framework, as part of a dedicated pathway for oral breast cancer therapies. The median age was 67 years (range 31–92 years). Most patients (n = 125, 95.4%) had an ECOG performance status of 0 or 1, and only 4.6% (n = 6) had an ECOG score of 2. Regarding histological tumor type, 82 patients (62.6%) had ductal carcinoma, 29 (22.1%) had lobular carcinoma, and 18 (13.7%) had non-specific ductal carcinoma. The endocrine therapy associated with palbociclib was mainly fulvestrant (48.9%) or letrozole (45.8%). Only 5.3% of patients received palbociclib in combination with other hormonal therapies (anastrozole or exemestane). The starting dose of palbociclib was 125 mg in 112 patients (85.5%), 100 mg in 16 patients (12.2%), and 75 mg in 3 patients (2.3%). The median six-month dose intensity was 15,800 mg, and the median follow-up was 32.4 months (range 5.4–51.8 months). At the six-month assessment, 10.7% of patients (n = 14) had progressive disease, 8.4% (n = 11) had a complete response, 35% (n = 46) had a partial response, and 60% (n = 60) had stable disease. At the time of data cut-off, 79% (n = 104) had experienced disease progression, with a median time to progression of 14.2 months (range 3.5–46.6 months).

### 3.2. Palbociclib Dose-Intensity Optimization

Forty-six patients (35.1%) received a non-optimized palbociclib dose intensity based on current recommendations (Group 1). Among them, 37 patients (80.4%) had one or more anticipated dose reductions (before or during the treatment course), and nine patients (19.6%) had one or more extended cycle delays. No patient experienced both an anticipated dose reduction and an extended cycle delay. Patient characteristics for the two groups are summarized in Table 2. There were no significant differences between the two groups in terms of cancer type, metastatic site, or associated endocrine therapy. However, significant differences were observed between the groups in terms of age, weight, ECOG performance status at treatment initiation, line of metastatic treatment, occurrence of grade ≥ 3 toxicity during the first three cycles, and palbociclib dose intensity. Group 1 patients were significantly older (median 74 vs. 64 years; *p* < 0.001), lighter (median 62 vs. 68 kg; *p* = 0.005), and had lower functional status (ECOG PS, *p* = 0.02) than Group 2 patients. Furthermore, the occurrence of grade ≥ 3 toxicity during the first three cycles (37.0% vs. 12.9%, *p* = 0.001) was significantly higher in Group 1 patients, and their palbociclib dose intensity was significantly lower.

### 3.3. Outcomes and Survival 

**Figure 1:**
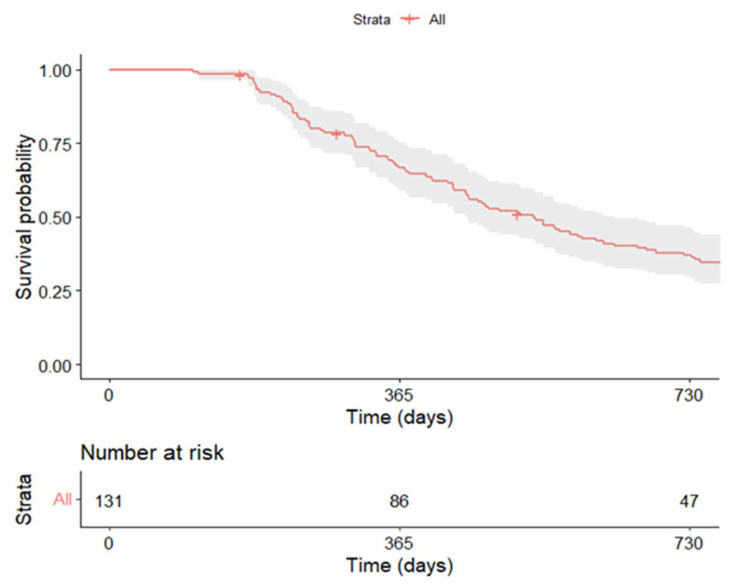
Kaplan–Meier plots of PFS in the overall population.

The median follow-up from treatment initiation was 30.7 months (range 5.5–51.8). During this period, 93 patients (71%) experienced disease recurrence, with a median time to progression of 14.2 months. Overall, 10.7% of patients (n = 14) showed disease progression at the six-month evaluation. In Group 2, only 2.4% (n = 2) of patients showed progression at six months compared with 26.1% (n = 12) in Group 1. The six-month disease control rate (complete or partial response or stable disease) was significantly higher in Group 2 (*p* < 0.001). Progression-free survival (PFS) for the overall population is shown in Figure 1. The median PFS was 17.5 months. 

**Figure 2:**
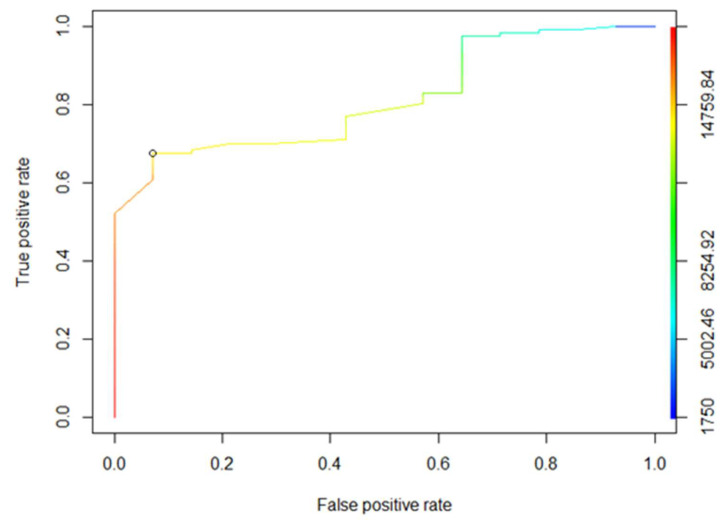
Receiver Operating Characteristic (ROC) analysis of 6-month dose intensity.

Receiver operating characteristic (ROC) analyses were performed to determine the threshold values of six-month dose intensity and the number of additional weeks of delay (beyond the recommendations) predictive of progression. The optimal cut-off value of 14,250 mg per six months was associated with a specificity of 68% and a sensitivity of 93% (Figure 2). The positive predictive value was 26%, and the negative predictive value was 99%. 

**Figure 3:**
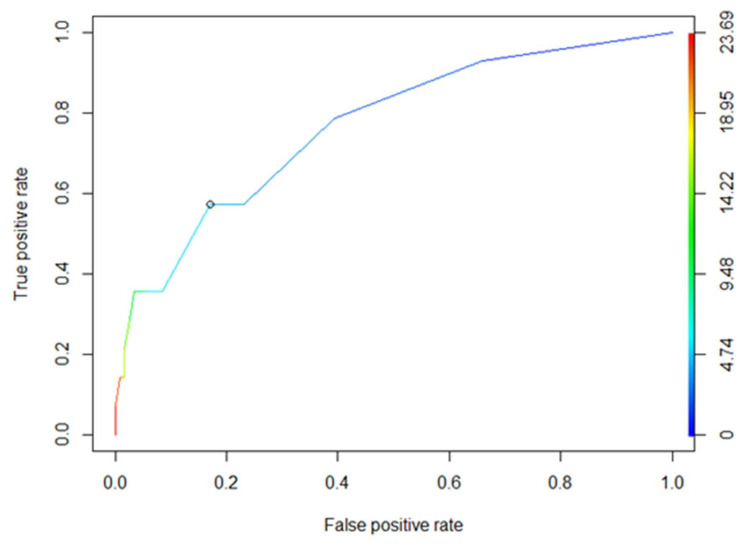
Receiver Operating Characteristic (ROC) analysis of cycle delays.

The optimal cut-off value for treatment delay was four weeks, corresponding to a specificity of 83% and a sensitivity of 57% (Figure 3). The positive predictive value was 29%, and the negative predictive value was 94%.

Univariate logistic regression analyses identified several factors correlated with six-month treatment response: non-optimized palbociclib dose intensity (OR = 14.6, 95% CI 3.74–97.4, *p* < 0.001), cycle delay > 4 weeks (OR = 5.94, 95% CI 1.58–21.0, *p* = 0.01), initial dose < 125 mg (OR = 4.09, 95% CI 1.13–13.7, *p* = 0.034), and six-month dose intensity < 14,250 mg (OR = 26.0, 95% CI 4.91–481, *p* < 0.001). Correlation analyses were used to assess the strength of the linear relationships between variables. As shown in Table 3, multivariate logistic regression identified cycle delays longer than four weeks as the only factor significantly increasing the risk of progression at six months. Baseline variables such as age, ECOG performance status, and line of metastatic treatment were evaluated in univariate analyses but were not retained in the final multivariate model due to the limited number of events.

**Figure 4:**
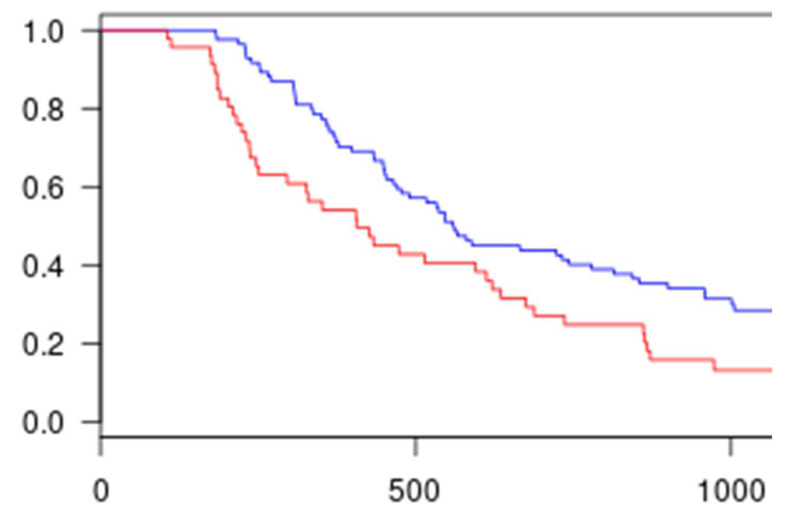
Kaplan–Meier plots of PFS in the population: group 2 (blue) and group 1 (red), *p* = 0.005.

The progression-free survival (PFS) analysis revealed a significantly longer PFS for Group 2 compared with Group 1 (Figure 4). Therefore, optimizing palbociclib dose intensity during the first six months of treatment was associated with a longer PFS in our study population. 

## 4. Discussion

The primary objective of this retrospective study was to evaluate the influence of palbociclib dose intensity on six months treatment response, and to assess the impact of dose reductions and cycle delays on progression-free survival (PFS). The emergence of new targeted therapies such as palbociclib has greatly improved the management of patients with hormone receptor-positive (HR+), endocrine-resistant metastatic breast cancer. Indeed, palbociclib has demonstrated statistically and clinically significant improvements in progression-free survival among patients with estrogen receptor-positive/human epidermal growth factor receptor 2-negative (ER+/HER2−) advanced breast cancer [11,12,13,14].

CDK4/6 inhibitors are generally well tolerated; however, adverse events—particularly hematologic toxicity—are common. In such cases, a palbociclib dose management algorithm is recommended by the pharmaceutical company to maintain the highest possible dose intensity. Nevertheless, dose reductions remain at the discretion of the treating physician when necessary to preserve patients’ quality of life or treatment adherence. Real-world data on reduced palbociclib dose intensity have rarely been reported. 

With a median PFS of 17.5 months, our study population showed a progression-free survival comparable to that reported in clinical trials, ranging from 9.5 to 27.5 months depending on patient characteristics such as age, line of metastatic treatment, and associated endocrine therapy [11,12,15,16].

Wilkie et al. reported PFS data in a real-world population, but excluded patients who initiated palbociclib at a reduced dose. In addition, cycle delays were not evaluated in their study [17]. The median PFS reported in real-world studies—including patients treated with palbociclib without restrictions regarding age, ECOG performance status, treatment line, associated endocrine therapy, or initial dose—ranges from 11.3 months to more than 24 months [18,19,20,21,22,23].

In our study, 35% of patients received a lower palbociclib dose intensity than recommended. Among this subgroup, 37 patients (80.4%) underwent an anticipated dose reduction—either at initiation (n = 19, 41.3%), during treatment (n = 5, 10.9%) or both (n = 13, 28.3%)—and 9 patients (19.6%) experienced extended cycle delays outside guideline recommendations. Patients with lower palbociclib dose intensity were significantly older, lighter, and had poorer functional status than those with optimized dose intensity. These patients also received a significantly higher median line of metastatic treatment. These findings suggest that physicians may adjust doses to preserve quality of life in clinically frail patients. However, no specific recommendations (such as early dose reduction or extended cycle delays) currently exist for these patients. On the contrary, PALOMA studies examining palbociclib use in elderly patients have shown that they are not at greater risk of toxicity than younger patients [16].

Previous studies have shown that individual dose reductions in palbociclib do not necessarily increase the risk of disease progression. However, our findings indicate that early dose reductions and extended cycle delays—particularly those longer than four weeks—are associated with an increased risk of progression [24,25]. Importantly, these associations must be interpreted in the context of confounding in the context of treatment-selection bias. Patients with dose reductions or cycle delays were older, had poorer performance status, more advanced treatment lines, and higher early toxicity rates, all of which are known to adversely influence prognosis. Dose modifications may therefore reflect underlying clinical frailty rather than being independent causal factors of disease progression.

With all the precautions warranted by the biases inherent to this study, we were able to identify several parameters that may influence the six-month treatment response in these patients, although these findings require confirmation in independent cohorts. Nevertheless, in our study population, each anticipated reduction of 100 mg in six-month dose intensity increased the risk of progression by approximately 3%. Patients who received a six-month dose intensity below 14,250 mg had a 26-fold higher risk of disease progression at six months. Our results also show that initiating treatment at a dose below 125 mg increased the six-month progression risk by 2.5-fold, and having a treatment delay longer than four weeks increased the six-month progression risk by four-fold. Therefore, a reduction in six-month dose intensity represents a risk factor for progression. Moreover, extended cycle delay appeared to have greatest impact on progression than anticipated dose reductions. These cut-offs were derived from ROC analyses performed within the same cohort and may therefore overestimate their predictive performance; external validation in independent cohorts is required before they can be applied in routine clinical practice. Nevertheless, these results may be useful in routine clinical practice to encourage early dose-intensity optimization in patients with good treatment tolerance. It should be noted that six-month progression was considered as an early binary outcome, while progression-free survival was analyzed separately using time-to-event methods. Given the retrospective nature of the study and the baseline heterogeneity between groups, these results should be interpreted with caution and viewed as associative rather than causal.

In this real-world study, we identified for the first time the impact of the six-month palbociclib dose intensity received by patients and, more specifically, the effects of anticipated dose reductions and extended cycle delays on six-month progression risk. We demonstrated that patients who experienced anticipated dose reductions or extended cycle delays had a lower palbociclib dose intensity during the first six months of treatment. Furthermore, patients with a non-optimized palbociclib dose intensity during the first six months experienced shorter progression-free survival (PFS). These survival results should, however, be interpreted with caution. Indeed, Group 1 patients presented additional risk factors for shorter survival—such as older age, poorer ECOG performance status, later lines of metastatic treatment, and higher rates of early severe hematologic toxicity—compared with Group 2 patients. Therefore, the two groups were difficult to compare rigorously. Our study also has several other limitations, including its retrospective, single-center design. Nevertheless, as a real-world study involving a relatively large number of patients, it provides useful insights into management factors associated with six-month progression.

Finally, specific patient characteristics may predispose individuals to an increased risk of early severe hematologic toxicity, thereby supporting the need of dose adjustments [25]. In selected cases, it would be valuable to assess palbociclib plasma concentrations to provide additional guidance for optimizing patient management.

## 5. Conclusions

Physicians sometimes need to adapt their patients’ treatment to preserve quality of life and maintain therapeutic adherence, even if this requires reducing dose intensity outside standard guidelines. By identifying management practices that may increase the six-month progression risk, this study offers guidance to help clinicians choose the most appropriate palbociclib management strategy for their patients while considering the risk of disease progression. Palbociclib remains a well-tolerated anticancer agent whose optimized dose intensity should be maintained as long as possible, keeping in mind each patient’s risk–benefit balance and the potential impact on progression-free survival.

## Figures and Tables

**Table 1:** Palbociclib dose modification and toxicity management according to the Summary of Product Characteristic (SmPC).

Toxicity	CTCAE Grade	Action
Hematologic toxicities	Grade 1–2	No action
	Grade 3 on Day 1	Withhold and repeat CBC within 1 week Once recovered to Grade ≤ 2: Resume at the same dose *
	Grade 3 on Day 15 of first 2 cycles	Continue at the same dose to complete cycle Repeat CBC on Day 22 If Grade 4 on Day 22, dose reduction guidelines *
	Grade 3 neutropenia with fever ≥ 38.5 °C and/or infection at any time Grade 4 at any time	Withhold Once recovered to Grade ≤ 2: Resume at next lower dose
Nonhematologic toxicities	Grade 1–2	No action
	Grade 3–4 persistent despite medical treatment	Withhold until grade ≤ 2 Resume at next lower dose

Abbreviations: CTCAE, Common Terminology Criteria for Adverse Events; CBC, Complete blood count. * Consider dose reduction in cases of prolonged (>1 week) recovery from Grade 3 neutropenia or recurrent Grade 3 neutropenia on Day 1 in subsequent cycles.

**Table 2:** Patient baseline demographic, clinical and biological characteristics in the two populations.

Characteristic	Group 1 Non Optimized Palbociclib Dose-Intensity n = 46	Group 2 Optimized Palbociclib Dose-Intensity n = 85	*p* Value ^§^
Age, median [IQR] years old	74 [67,80]	64 [55,69]	<0.001
Weight, median [IQR], kg	62 (54,70)	68 (62,78)	0.05
ECOG performance status, n (%)			
0	12 (26.1)	35 (41.2)	0.02
1	15 (32.6)	32 (37.6)	
2	5 (10.9)	1 (1.2)	
Unknow	14 (30.4)	17 (20.0)	
Cancer type, n (%)			
Ductal Non specific	5 (10.9)	13 (15.3)	0.6
Ductal	29 (63.0)	54 (63.5)	
Lobular	11 (23.9)	18 (21.2)	
Unknow	1 (2.2)	0 (0)	
Disease site, n (%)			
Visceral	28 (60.9)	49 (57.6)	0.7
Node	21 (45.7)	46 (54.1)	0.4
Bone	36 (78.3)	69 (81.2)	0.7
Metastatic treatment line, median [IQR]	2 [1,2]	1 [1,2]	0.03
Initial dosage palbociclib, n (%)			125 mg vs. other <0.0001
125 mg	27 (58.7)	85 (100)	
100 mg	16 (34.8)	0 (0)	
75 mg	3 (6.5)	0 (0)	
Hormonal therapy associated, n (%)			*Fulvestrant* vs. *Other* 0.86
Fulvestrant	22 (47.8)	42 (49.4)	
Letrozole	22 (47.8)	38 (44.7)	
Other	2 (4.4)	5 (5.9)	
Hematotoxicity ≥ Grade 3 during the first three cycles, n (%)			
Yes	17 (37.0)	11 (12.9)	
No	29 (63.0)	74 (87.1)	0.014
6-month dose-intensity/100, median [IQR], mg	117 [89,133]	168 [158,174]	<0.001
Progression at 6-month evaluation, n (%)			
Yes	12 (26.1)	2 (2.4)	
No	34 (74.9)	83 (97.6)	<0.001

^§^ The data were evaluated with Pearson’s χ^2^ test or Fisher’s exact test when appropriate and Wilcoxon rank sum test. IQR, interquartile range.

**Table 3:** Multivariate analysis of predictive factors.

Variables	Final LR Model		
	OR	95% CI	*p* Value
Cycle delay > 4 weeks (Yes vs. No)	4.79	1.20–17.7	0.028
Palbociclib initial dosage (125 mg vs. other)	3.13	0.79–11.1	0.10

Abbreviations: LR, logistic regression; OR, Odds Ration; 95% CI, 95% Confidence Interval.

## Data Availability

The data presented in this study are available on request from the corresponding author.
